# Examining the Dose-Response Effects of Mindfulness Meditation Interventions on Well-Being: Protocol for a Randomized Controlled Trial

**DOI:** 10.2196/72786

**Published:** 2025-07-29

**Authors:** Nicholas Bowles, Alexander Burger, Jonathan N Davies, Julie A Simpson, Julieta Galante, Simon Dennis, Benjamin Stone, Nicholas T Van Dam

**Affiliations:** 1 Melbourne School of Psychological Sciences The University of Melbourne Melbourne Australia

**Keywords:** mindfulness, meditation, dose-response, well-being, internet- and mobile-based interventions

## Abstract

**Background:**

Mindfulness meditation has demonstrated modest benefits for mental health and well-being, although the relationship between practice dose and outcomes is unclear. Meta-analyses and randomized controlled trials have shown mixed results so far, although such results may stem from methodological issues rather than reflecting the absence of an underlying effect. Research outside structured programs suggests that long-term practice time is linked to positive outcomes, but bias due to self-selection over time may explain these results.

**Objective:**

The proposed trial aims to test dose-response effects for an online mindfulness meditation course, examining outcomes and participant engagement across different practice doses. In this pragmatic randomized controlled trial, we hypothesize that larger doses of mindfulness training will yield significantly larger effects and different doses will be significantly associated with variation in participant engagement, with lower engagement evident for higher doses.

**Methods:**

At least 688 healthy adults aged between 18 and 65 years will be randomized to join one of three 4-week online mindfulness courses with daily practices of varying lengths (ie, 10, 20, or 30 min) against a minimally active control condition (4 min). Psychological well-being will be measured using the Warwick-Edinburgh Mental Wellbeing Scale at the baseline, midintervention, and postintervention time points and at 1-month follow-up. Secondary outcomes are psychological distress, anxiety, depression, social anxiety, nonattachment, trait mindfulness, decentering, equanimity, repetitive negative thoughts, emotion regulation, attention control, and emotional reactivity. Other outcomes will be collected weekly and daily during the intervention period. The primary analysis will be undertaken following the intention-to-treat approach. We will also conduct per-protocol secondary analyses on all outcomes (ie, primary and secondary). In addition, we will systematically monitor for possible adverse experiences.

**Results:**

This study began screening and recruitment in May 2024. Recruitment was paused approximately 6 weeks later after a substantial number of participants were identified as being fraudulent and not meeting the eligibility criteria. Recruitment reopened in October 2024, and by the end of 2024, a total of 70 eligible participants were enrolled. Recruitment recommenced in early 2025 and will continue until the end of March 2025 or until the target sample is reached. We estimate that the results will be published by March 2026.

**Conclusions:**

This study will contribute to the evidence base for mindfulness meditation and the question of how much practice people need to engage in to improve well-being and other psychological outcomes.

**International Registered Report Identifier (IRRID):**

DERR1-10.2196/72786

## Introduction

### Background and Rationale

There is now reasonable evidence that mindfulness meditation can have modest beneficial effects on mental health and well-being [1-4]. For an individual starting to practice mindfulness meditation with the objective of improving their mental health and well-being, a natural question is how much practice is needed to achieve certain benefits. The empirical evidence that can be brought to bear on this question is still in its infancy. While benefits have been demonstrated at various practice intensities, there is little evidence of how different doses of daily practice relate to outcomes and whether and to what degree more practice is likely to deliver greater outcomes. Of the 4 empirical studies that have experimentally manipulated session duration to assess dose-response effects for mental health and well-being outcomes, most have failed to detect dose-response effects. However, the authors noted limited dose variation and program duration as limitations, with each of the studies including only 2 dose conditions ranging from 5 to 30 minutes in programs lasting up to 2 weeks of daily practice [5-8]. Meta-analyses investigating dose-response effects in which dose has been defined by the daily home practice component of mindfulness-based programs (MBPs) have so far shown mixed results. A meta-analysis by Parsons et al [9] found a small correlation between self-reported home practice and psychological conditions such as anxiety, depression, and stress (*r*=0.26, 95% CI 0.19-0.34), but a more recent meta-regression from Strohmaier [10] found no statistically significant dose-response effects for the same psychological conditions (where dose was defined as both recommended and self-reported home practice). However, as argued in the study by Bowles et al [11], the absence of evidence for daily practice dose-response effects may not constitute evidence of their absence and may, instead, be due to methodological issues. For instance, as daily practice dosages have not been experimentally manipulated in randomized designs [10], causation about the effects of different dosages cannot be inferred. In addition, as described in the study by Bowles et al [11], given that such meta-analyses were conducted on effect sizes that relate to overall program content, which features multiple active and mutually reinforcing elements [12], it is not known to what extent variation in daily practice time contributes to overall outcomes. Therefore, these dose-response meta-regression models may lack the sensitivity to detect the effects of marginal differences in daily home practice dose and, thus, may be ill-equipped to inform the question of the optimal amount of practice.

Outside the context of MBPs, regular mindfulness meditation dose-response investigations have focused primarily on the relationship between cumulative practice time over the course of an intervention and outcomes. In experimental conditions, such variation in practice time has arisen from natural differences across participants rather than systematic manipulations. While most [13-15], though not all [16], studies support a positive association between time spent practicing and outcomes, such findings may be subject to bias due to the nonrandom distribution of participants across different practice doses. In low-dose settings, while benefits have been demonstrated from single short sessions approximating 15 minutes [17], the measures used in such studies have tended to focus on transient or *state* effects that are more sensitive to change. Therefore, it is not clear what minimum dose is required for efficacious practice when measures focus on trait effects related to the psychological domains that most often motivate practice [18]. In addition, while it is likely that participants in high-dose settings such as meditation retreats represent individuals who derived enduring benefits from the practice, as evidenced by exceptional traits in several psychological and cognitive domains [19], such results may be biased by self-selection [20]. Therefore, there is a lack of empirical work in which practice doses are systematically varied in such a way as to enable differential effects to be causally linked to different practice doses. Moreover, because of this, there is a concomitant lack of empirical evidence of the merits of different practice doses given an individual’s circumstances and disposition and knowledge of the trade-offs from recommending higher and lower doses.

### Objectives

The proposed trial aims to (1) test for a dose-response effect on well-being across the active conditions (which feature different doses of mindfulness meditation instructions and practices; 10, 20, and 30 min) and a minimally active control condition (ie, minimal dose <10 min, <20 min, and <30 min) and (2) explore whether differential rates of program engagement and retention are evident across the 4 (active and control) conditions.

Our hypotheses are that (1) larger doses of mindfulness training will yield significantly larger effects on well-being and (2) different doses will be significantly associated with variation in participant engagement, with lower engagement evident for higher doses.

## Methods

### Trial Design

#### Overview

**Figure 1:**
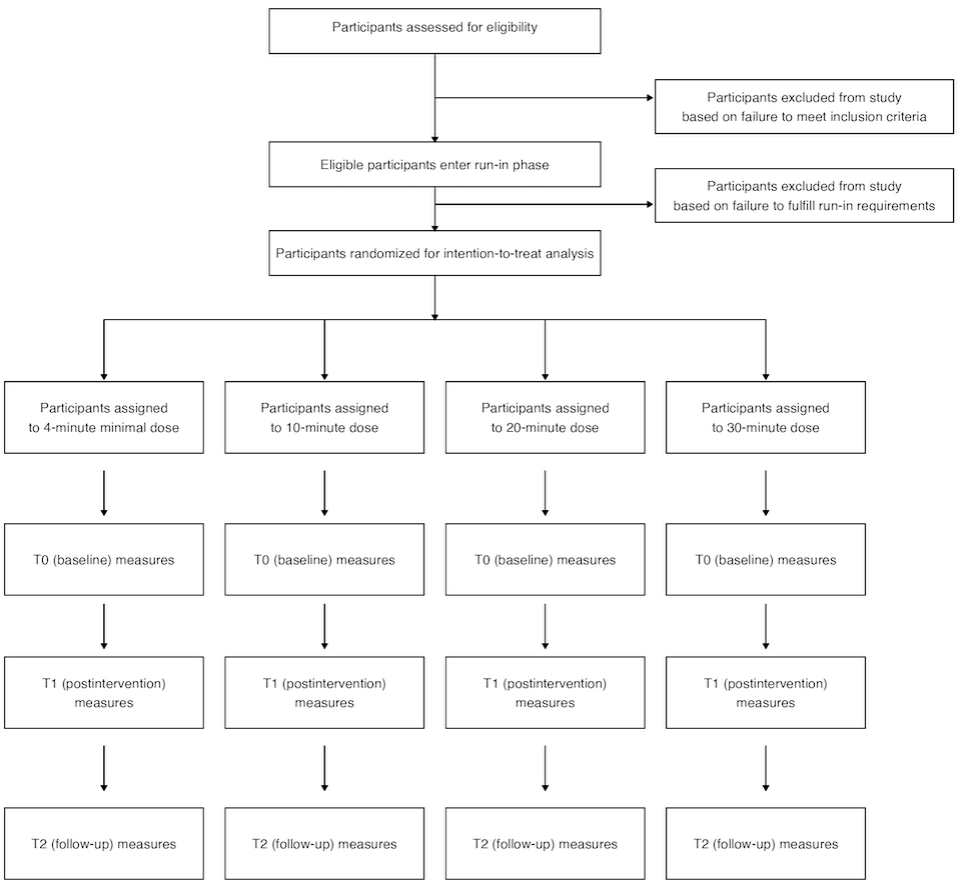
CONSORT (Consolidated Standards of Reporting Trials) flow diagram.

This study will compare the efficacy of receiving different “doses” of mindfulness training over a 28-day period in a prospective 4-arm, parallel-group randomized controlled trial. Participants will be randomized into 1 of the 4 groups: 10 minutes of meditation per day, 20 minutes of meditation per day, 30 minutes of meditation per day, or a minimal dose of 4 minutes of meditation per day (Figure 1). All participants will be encouraged not to complete any further mindfulness training or practice during the intervention period apart from informal practice during daily life. We chose these session lengths for our dose conditions to align with the range of dose variations that have been used in previous experimental research [5-8], as well as our own review of expert meditation teachers, as outlined by Bowles et al [11].

Self-reported biopsychosocial measures will be evaluated at 4 time points: before randomization (baseline; T0), 2 weeks after randomization (midintervention time point; T0.5), 4 weeks after randomization (postintervention time point; T1), and 8 weeks after randomization (follow-up; T2). In addition, weekly outcomes will be evaluated between T0 and T1. Individual adherence and meditation-related experiences (including adverse events) will be monitored and compared across treatments. The trial will consist of 3 main phases: the run-in period, the intervention period, and the follow-up period, as described in the following sections.

#### Run-In Period

We will randomize after the completion of a 7-day run-in period. Estimates of mindfulness and meditation app user retention over 28 days indicate that most attrition (approximately 90%) occurs within the first 7 days of app installation [21]. Therefore, the run-in period will be used to select the participants most likely to complete study measures. During the run-in period, we will ask participants to complete daily measures (refer to the Outcomes section) as well as listen to four short (5 min) “preparation for meditation” audios covering the following topics: (1) background of the teacher and mindfulness, (2) introduction to the concept of mindfulness, (3) introduction to mindfulness practice, and (4) results from practicing mindfulness. All this content will be accessed via a series of Qualtrics surveys (Qualtrics International Inc) that will be emailed to participants daily. Participants will also be asked to respond to measures that cover personality, sleep, and motivations and expectations. Participants will have successfully completed the run-in period if they engage with ≥57% (ie, at least 4 of the 7 days) of the run-in content.

We will monitor participant response patterns during the run-in period. If such monitoring raises suspicions about the accuracy of the information provided in the prescreening survey (ie, related to the eligibility criteria—most notably, participant identity and country of residence), additional verification procedures may be required. Participants flagged as suspicious who fail to complete or successfully pass additional verification procedures (refer to the Eligibility Criteria section) will be excluded from the study.

#### Intervention Period

Participants who successfully complete the run-in period will be first asked to complete outcome measures at T0 (baseline) via Qualtrics. After completing outcome measures, participants will be randomized to 1 of the 4 study groups and provided with a unique participant identification code along with instructions on how to access the intervention materials. Participants will be asked to use only the specific mindfulness instructions provided for 28 days and complete all survey items when prompted. Throughout the 28-day period and at the conclusion of the intervention, participants will be prompted to complete outcome measures as specified in the Outcomes section.

#### Follow-Up Period

At the conclusion of the 28-day intervention period, participants will be offered full access to any of the 3 higher-dose courses that they choose. At the conclusion of the follow-up period (1 month after completion of the intervention), participants will again complete outcome measures as specified in the Outcomes section and Figure 1.

### Study Setting

This study will be conducted entirely online; participants will complete the intervention (mindfulness program) in real-world (and, thus, ecologically valid) settings. Participants will be recruited and will communicate with the research team online. All data collection will be completed via their mobile phone, PC, or tablet.

### Eligibility Criteria

Participants who provide written informed consent and meet the inclusion and exclusion criteria provided in Textbox 1 will be eligible for the study.

**Inclusion and exclusion criteria.**

**Inclusion criteria**

Age of 18 to 64 years (working-age adults as specified by the Australian Bureau of Statistics)
Residence in Australia, with no overseas relocation or travel plans that would impact the ability to participate in the study (ie, engage with meditation practices or receive and respond to surveys)
Sufficient comprehension of the English language to complete measures
Registration for an account and agreement to the terms and conditions of Unforgettable.me, a web platform administered by Unforgettable Research Services Pty Ltd
If experiencing anxious or depressive symptoms of moderate severity (defined by a score of >19 on the Patient-Reported Outcomes Measurement Information System [PROMIS] Anxiety Short Form or >22 on the PROMIS Depression Short Form), agreement to continue the recommended routine medical treatment for eligible underlying mental or physical health conditions for the duration of the study and to seek additional treatment if indicated by deterioration of symptoms
Successful completion of the run-in period
No or minimal experience with meditation, defined as <25 hours over the previous 6 months, and having never attended a multiday mindfulness course (eg, mindfulness-based stress reduction or Vipassana meditation). If we have not recruited 50% of our target sample size within the first 3 months of active recruitment, we will broaden the inclusion criteria to participants with up to 100 hours of mediation experience in the previous 6 months

**Exclusion criteria**

- Self-reported current or lifetime serious mental illness (eg, neurodevelopmental, schizophrenia spectrum, bipolar, obsessive-compulsive, trauma-related, dissociative, or personality disorder)
- Anxious and depressive symptoms in the severe range (defined by a score of >27 on the PROMIS Anxiety Short Form or >32 on the PROMIS Depression Short Form)
- Psychological distress symptoms in the severe range (Kessler Psychological Distress Scale ≥30)
- Threshold scores on prescreening and follow-up measures exceeded for mania, suicidal ideation, psychosis, repetitive thoughts and behaviors, and dissociation
- Moderate or higher alcohol, tobacco, or drug use
- Self-reported diagnosis of a neurological condition (eg, traumatic brain injury, amnesia, epilepsy, or stroke)
- Self-reported presence of any serious medical condition (eg, cancer, thyroid disorder, or multiple sclerosis)
- Recent bereavement or major loss
- History of unexplored, untreated traumatic experiences or adverse childhood events

The selection criteria will be self-assessed. Throughout the study, we will conduct various checks to verify the accuracy of the information provided in the prescreening survey to determine study eligibility. Participants whom we suspect provided inaccurate information may be required to verify specific details. If we cannot verify such information within 1 week, that participant will be deemed to have responded inaccurately and excluded from the study. Although we will make every effort to flag and remove inauthentic participants before randomization (ie, during the run-in period), in some cases, it may not be possible and, therefore, removal will take place after randomization. The Assignment of Interventions: Allocation and Blinding section provides details on how we will handle postrandomization exclusion.

Details on the procedure that we will use to flag and remove inauthentic participants were preregistered but are being kept confidential from study participants to avoid influencing behavior or actions that could compromise the procedure. Accordingly, the exact methodology has been embargoed [22] and will only be disclosed after data collection closes.

### Prescreening Measures

Individuals will complete a prescreening survey to determine their eligibility.

*Sociodemographic* questions will consist of items related to age and gender. Supplementary sociodemographic questions will be asked on day 1 of the run-in period, comprising questions about ethnicity, household status, indigenous status, and education status.

*English proficiency* will be assessed by asking whether the primary language spoken is English. Participants for whom English is not their primary language will be asked whether they have sufficient proficiency to complete the study given that all study materials are in English.

*Residency and location* during the study will be assessed by asking whether the participant resides in Australia; whether they intend to travel outside Australia during the following 5 weeks; and, if so, whether such travel is likely to affect their participation.

*Mental health status* will be measured using the *Diagnostic and Statistical Manual of Mental Disorders, Fifth Edition*, Self-Rated Level 1 Cross-Cutting Symptom Measure [23], a 23-item self-report measure designed to assess symptoms across 13 domains of mental health. We will measure 5 of the 13 domains: mania, suicidal ideation, psychosis, repetitive thoughts and behaviors, and dissociation. We will administer level 2 follow-up measures (Table 1) in cases in which scores for any level 1 domain exceed clinical cutoffs. For depression and anxiety, level 2 measures will be administered in lieu of the relevant level 1 items. Additional screening measures will include (1) the Kessler Psychological Distress Scale, for anxiety and depressive symptoms; (2) a single-item measure of substance use within the last year that, if any item is endorsed, will trigger a more detailed drug and alcohol screening questionnaire; and (3) a single-item measure of trauma that, if endorsed, will trigger a more detailed trauma screening questionnaire. Endorsement of any item on the more detailed screener will trigger a brief questionnaire to screen for posttraumatic stress disorder.

*Meditation history* will ask individuals whether they have practiced meditation in the past and, if so, to select the types of meditation they have practiced and total estimated practice time in the previous 6 months and since commencing.

The other selection criteria will be determined by asking participants directly (eg, a question about recent bereavement).

**Table 1:** Follow-ups to prescreening measures if the threshold score is exceeded.

Domain of interest	Measure	Criteria	Description
Mania	ASRM^a^	Raw score>10^b^, indicating high probability of manic or hypomanic condition	The ASRM is a 5-item measure that assesses mood, self-confidence, sleep disturbance, speech, and activity level [24].
Suicidal ideation	DSI-SS^c^	Total score>2	The DSI-SS is a 4-item measure that assesses the frequency and intensity of suicidal ideation, formulation of plans for suicide, the controllability of suicidal thoughts, and impulses related to suicide.
Psychosis	PQ-16^d^	Total score>5	The PQ-16 is a 16-item measure of psychosis with 9 items on perceptual abnormalities and hallucinations, 5 items on unusual thought content, and 2 items on negative symptoms.
Repetitive thoughts and behaviors	Repetitive Thoughts and Behavior	Average score of >2 (total raw score ≥11), indicating severe or extreme symptoms	Repetitive Thoughts and Behavior is a 5-item assessment of repetitive thoughts and behaviors that has been adapted from the FOCI^e^ Severity Scale.
Dissociation	DES-B^f^-Modified	Average score of >2 (total raw score ≥17), indicating severe or extreme symptoms	The DES-B-Modified is an 8-item assessment of the severity of dissociative experiences [25].
Substance use	NIDA^g^ Quick Screen (version 1.0), NIDA-Modified ASSIST^h^, and AUDIT^i^ alcohol screening tool	See the “Description” column	We will ask participants whether they have used alcohol (≥5 drinks for men and ≥4 drinks for women), tobacco, prescription drugs for nonmedical reasons, or other illegal drugs in the past year. If the participant endorses “once or twice” or more frequent use in the last year, we will ask which drugs and additionally ask about frequency of use in the past 3 months (“never,” “once or twice,” “monthly,” “weekly,” “daily,” or “almost daily”). For alcohol, participants reporting daily use (of 5 drinks for men and 4 drinks for women) will be excluded. Participants reporting weekly or monthly use will be administered the alcohol screening tool, and a score of ≥14 will result in the participant being excluded. For tobacco, participants reporting daily use will be excluded. For drugs (prescription drugs for nonmedical reasons or illegal drugs), participants reporting weekly or daily use will be excluded. If participants report monthly use or use once or twice, we will ask additional questions as follows: (1) “In the past three months, how often have you had a strong desire or urge to use [drug]? How often has your use of [drug] led to health, social, legal, or financial problems? How often have you failed to do what was normally expected of you because of your use of [drug]?” (2) “Have you ever tried and failed to control, cut down, or stop using [drug]?” (response options: “no” and “yes”). For drugs other than cannabis, if monthly or more frequent use is reported for any of the 3 items from question 1 or the participant answers “yes” within the last 3 months for question 2, they will be excluded. For cannabis, if weekly or more frequent use is reported for any of the 3 items from question 1 or the participant answers “yes” within the last 3 months for question 2, they will be excluded.
Trauma	Single-item measure of trauma history and BTQ^j^	The BTQ is administered if the item is endorsed. Endorsement of any item on the BTQ will trigger assessment of PTSD^k^ (see the following row)	We will ask participants to respond to the following item: “Sometimes things happen to people that are unusually or especially frightening, horrible, or traumatic. For example, a serious accident or fire; a physical or sexual assault or abuse; an earthquake or flood; seeing someone be killed or seriously injured; having a loved one die through homicide or suicide. Have you ever experienced this kind of event?” (response options: “yes” and “no”). If the participant answers “yes,” the BTQ will be administered. The BTQ is a 10-item measure to ascertain whether the respondent has experienced a traumatic event, which includes experiences of potentially traumatic events such as crime and sexual and physical assault [26]. Endorsement of any item on the BTQ will trigger an assessment of PTSD (refer to the following row).
PTSD	PCL-5A^l^	Endorsement of any item indicates probable PTSD	The PCL-5A is a 5-item measure used to screen for the presence of PTSD.

^a^ASRM: Altman Self-Rating Mania Scale.

^b^The exclusion threshold for the ASRM was updated from >5 to >10 as of March 12, 2025, after it became apparent that the original threshold was too sensitive, excluding a higher-than-expected number of participants. A subsequent review of the literature confirmed that the ASRM has low specificity in nonclinical populations [27], supporting the decision to revise the threshold. The updated threshold was applied retrospectively, and individuals who had been excluded under the previous criterion but scored ≤10 were invited to recomplete the prescreening questionnaire.

^c^DSI-SS: Depressive Symptom Index–Suicidality Subscale.

^d^PQ-16: 16-item Prodromal Questionnaire.

^e^FOCI: Florida Obsessive-Compulsive Inventory.

^f^DES-B: Brief Dissociative Experiences Scale.

^g^NIDA: National Institute on Drug Abuse.

^h^ASSIST: Alcohol, Smoking, and Substance Involvement Screening Test.

^i^AUDIT: Alcohol Use Disorders Identification Test.

^j^BTQ: Brief Trauma Questionnaire.

^k^PTSD: posttraumatic stress disorder.

^l^PCL-5A: abbreviated Posttraumatic Stress Disorder Checklist for the Diagnostic and Statistical Manual of Mental Disorders, Fifth Edition.

### Interventions

#### Active Condition With Varied Doses

This study will include 3 active conditions that are dose variants of a newly developed 28-day mindfulness meditation program. The program is based on the *Satipatthana Sutta* (*The Discourse on the Establishing of Mindfulness* [28]—a foundational text within the Vipassana or insight tradition of Theravada Buddhism on which MBPs such as mindfulness-based stress reduction are largely based [29]). Most empirical work in the field of meditation has been conducted in the context of MBPs. The program generally aligns with the definition of an MBP developed by Crane et al [30]. The *Satipatthana Sutta* is structured into 4 progressive “contemplations”: body, feelings, mind, and “dhammas” or mental qualities [28]. This program will feature daily instructions and guided practices that are based on the first 3 contemplations: the body, feelings, and the mind.

The program has been developed independently by Australian meditation teacher Patrick Kearney with support from Australian meditation teacher Jess Huon based on specifications (ie, number of sessions, session length and duration, and intended audience) but with limited additional input from the research team. Kearney has practiced meditation within the insight tradition since 1977 and has been teaching meditation full time for the past 20 years.

All 3 variants of the program will feature the same structure, progression, and content but will vary in length of daily teaching and practice: 10, 20, and 30 minutes. Each program consists of an introduction, practice, and conclusion. While the introduction and conclusion are consistent across the 3 programs, longer sessions include more practice points. Additional practice points in the longer sessions represent elaborations on content already introduced rather than new themes. In total, each 10-minute session includes 5 practice points, each 20-minute session includes 10 practice points, and each 30-minute session includes 15 practice points. The proportion of time allocated to spoken content varies across the 3 programs accordingly, from 62% for the 10-minute sessions to 47% for the 20-minute sessions and 40% for the 30-minute sessions.

Practices will be provided for 6 days each week (ie, a total of 24 guided practices), with participants given the option to practice without guidance on the seventh day. Each variant will teach the same core content; however, the longer variants will have more time to elaborate on this content through teaching and gain experience practicing the guided techniques.

Practice instructions will consist of a brief explanation of relevant theory as well as a set of instructions for the practice in the first few minutes, followed by intermittent guided instructions throughout the duration of the practice. The programs will consist entirely of prerecorded material and will be delivered asynchronously. Therefore, there will be no direct contact between teachers and participants for the duration of the program. While this element constitutes a point of departure from how most MBPs are delivered, Crane et al [30] note that the exact method of delivery is flexible and can vary across MBPs [31].

After the 28-day intervention period, participants will be offered full access to any of the 3 higher-dose courses that they choose. During this time, participants will be free to choose to engage as much or as little with the guided practices as they wish.

#### Minimal-Dose Control Condition

The minimal-dose condition will feature a short daily meditation approximating 4 minutes (akin to the breathing space practice in mindfulness-based cognitive therapy or short practices in popular meditation apps). This condition is intended to provide a brief introduction to the foundations of mindfulness in theory and practice and follows the same course structure as the 3 active conditions. However, due to time constraints, the content will be very superficial.

### Outcomes

#### Overview

*Personality* will be measured on days 2 and 3 of the run-in period using the short form of the Big Five Inventory, a 30-item measure of personality across 5 domains: open-mindedness, conscientiousness, extraversion, agreeableness, and negative emotionality [32].

*Sleep* will be assessed on day 4 of the run-in period using a single item from the Sleep Quality Scale [33]: “During the past week, how would you rate your sleep quality overall?” (response options: *terrible*=1, *poor*=2-4, *fair*=5-7, *good*=7-9, and *excellent*=10).

*Motivations and expectancy* will be measured on day 4 of the run-in period using the following questions: (1) “How much benefit do you expect from this mindfulness meditation course?” (response options: not at all to very much), (2) “What is your main goal for completing this mindfulness meditation course?” (goal or motivation free-text field), (3) “How motivated are you by this goal?” (response options: not at all to very much), (4) “How much improvement or progress toward your goal do you expect from the course?” (response options: none at all to a great deal), and (5) “How confident are you that the amount of improvement or progress you indicated will occur?” (response options: not confident at all to extremely confident).

The outcome measures in the following paragraphs will be obtained at baseline (T0) and the midintervention (T0.5) and postintervention (T1) time points and after a 1-month follow-up period (T2) unless otherwise indicated.

*Psychological well-being (primary outcome)* will be assessed at T0, T1, and T2 using the Warwick-Edinburgh Mental Wellbeing Scale (WEMWBS) [34], which includes 14 positively worded items of mental well-being for the previous 2 weeks on a 5-point Likert scale from 1 (none of the time) to 5 (all of the time). The primary outcome measurement is at T1. Previous analyses of responsiveness to change analyses indicate that the WEMWBS detects individually meaningful improvement across a variety of mental health measures [35]. These analyses indicated an average SE of measurement over time of 2.69.

*Psychological distress (secondary outcome)* will be assessed at T0, T0.5, T1, and T2 using the Kessler Psychological Distress Scale [36], a 10-item self-report measure of psychological distress over the previous month, on a 5-point Likert scale from 1 (none of the time) to 5 (all of the time).

*Anxiety (secondary outcome)* will be assessed at T0, T0.5, T1, and T2 using the Patient-Reported Outcomes Measurement Information System (PROMIS) Anxiety Short Form [37], a 7-item measure of the pure domain of anxiety over the previous 7 days, on a 5-point Likert scale from 1 (never) to 5 (always) and the anxiety subscale of the Depression, Anxiety, and Stress Scale [38], a 7-item measure of anxiety over the previous 7 days that is assessed on a 4-point Likert scale from 0 (did not apply to me at all—never) to 3 (applied to me very much or most of the time).

*Depression (secondary outcome)* will be assessed at T0, T0.5, T1, and T2 using the PROMIS Depression Short Form [37], an 8-item measure of depression over the previous 7 days, on a 5-point Likert scale from 1 (never) to 5 (always).

*Social anxiety (secondary outcome)* will be assessed at T0 and T1 using the Social Anxiety Disorder Severity Scale [39], a 10-item measure of the severity of symptoms of social anxiety (phobia) in the previous 7 days, on a 5-point Likert scale from 0 (never) to 4 (all of the time).

*Nonattachment (secondary outcome)* will be assessed at T0, T0.5, T1, and T2 using the Nonattachment Scale [40], which measures the Buddhist concept of nonattachment on a 5-point Likert scale from 1 (disagree strongly) to 5 (agree strongly).

*Trait mindfulness (secondary outcome)* will be assessed at T0, T0.5, T1, and T2 using the Five Facet Mindfulness Questionnaire [41,42], a 15-item measure that includes 5 factors representing elements of mindfulness, on a 5-point Likert scale from 1 (never or very rarely true) to 5 (very often or always true).

*Decentering (secondary outcome)* will be assessed at T0, T0.5, T1, and T2 using the Experiences Questionnaire [43,44], an 11-item measure of decentering, on a 5-point Likert scale from 1 (never) to 5 (all the time).

*Equanimity (secondary outcome)* will be assessed at T0, T0.5, T1, and T2 using the Two-Factor Equanimity Scale [45], a 14-item measure of equanimity, on a 5-point Likert scale from 1 (never) to 5 (very often or always).

*Repetitive negative thoughts (secondary outcome)* will be assessed at T0, T0.5, T1, and T2 using the Repetitive Negative Thoughts Questionnaire [46], a 22-item measure of repetitive negative thinking, on a 6-point Likert scale from 1 (not at all typical of me) to 6 (extremely typical of me).

*Emotion regulation (secondary outcome)* will be assessed at T0, T0.5, T1, and T2 using the Difficulties in Emotion Regulation Scale–Short Form [47], an 18-item measure that includes 6 factors representing emotional regulation problems, on a 5-point Likert scale ranging from 1 (almost never) to 5 (almost always).

*Attention control (secondary outcome)* will be assessed at T0, T0.5, T1, and T2 using the Attention Control Scale–Short Form [48], a 10-item measure that includes 2 subscales of attention (shifting and focusing), on a 4-point Likert scale ranging from 1 (almost never) to 4 (always).

*Emotional reactivity (secondary outcome)* will be assessed at T0, T0.5, T1, and T2 using the Perth Emotional Reactivity Scale–Short Form [49], an 18-item measure of the activation, intensity, and duration of one’s emotional responses, on a 5-point Likert scale from 1 (very unlike me) to 5 (very like me).

#### Weekly Measures

During the intervention period, additional measures will be obtained weekly (ie, 7, 14, 21, and 28 days after the baseline survey).

*Mindfulness* practice will be assessed using the following items: (1) “In the past week, how many daily sessions of this study’s meditation program did you complete?” (response options: 0-7); (2) “In the past week, on the days you meditated with the study program, approximately how many minutes (on average) was each meditation session?”; (3) “In the past week, how many additional days did you practice meditation?” (response options: 0-7) and “In the past week, on the days you meditated in addition to the study program, approximately how many minutes (on average) was each meditation session?”; (4) “On the days you practised meditation, how many minutes per session did you practice? (this includes the guided sessions in the meditation program)” (response options: 0 to ≥60 min); and (5) “In the past week, how often did you engage in informal mindfulness practice? i.e. mindfully washing dishes, showering, walking etc.” (response options: none of the time to all of the time).

*Adverse events* will be assessed using a modified short form of the Meditation-Related Adverse Effects Scale [50].

*Sleep* will be assessed using a single item from the Sleep Quality Scale [33]: “During the past week, how would you rate your sleep quality overall?” (response options: *terrible*=1, *poor*=2-4, *fair*=5-7, good=7-9, and *excellent*=10).

*Expectancy* will be assessed using the following item: “How effective do you think your training so far has been for your [goal or motivation free-text field from run-in survey]?” (response options: not at all effective to very effective).

#### Momentary Measures

Participants will be assessed on study variables *daily*, before and after each meditation practice session. Meditators will respond to questionnaires assessing momentary mood before and after each meditation. Following the postmeditation mood items, meditators will complete 1 random questionnaire from a list of questionnaires assessing state mindfulness, decentering, mindful attitudes (including nonjudgment, nonattachment, and curiosity), and attention (attention regulation and self-regulation).

Participants who choose to skip a meditation session will be queried about their momentary mood and assessed on 1 random questionnaire from a list of questionnaires assessing disposition mindfulness, decentering, and attention control variables in the previous 24 hours.

*Momentary mood* will be assessed daily, before and after each meditation session, using a modified English version of the Multidimensional Mood Questionnaire [51]. The Multidimensional Mood Questionnaire assesses valence, arousal, and calmness dimensions of momentary mood using 6 items on a 7-point Likert scale (with the end points labeled as “very”). Participants will respond to the following statement—“At this moment I feel:”—with the following options: (1) tired-awake, (2) content-discontent, (3) agitated-calm, (4) energetic–lacking energy, (5) well-unwell, and (6) relaxed-tense.

*State mindfulness* will be assessed using a brief 6-item version of the State Mindfulness Scale [52]. The chosen items are among the highest-loading items on the original scale. Participants will respond to the following statement—“Please use the rating scale to indicate how well each statement describes your experience during the meditation:”—on a 5-point Likert scale with options ranging from 1 (not at all) to 5 (very well).

*Attention* will be assessed using the attention regulation and self-regulation subscales of the Multidimensional Assessment of Interoceptive Awareness [53] scale. A selection of 9 items based on the highest-loading items on the original subscales will be used to capture attention within the meditation session [53]. Participants will respond to the following statement—“Please indicate how often each statement applied to you during the meditation:”—on a 6-point Likert scale ranging from 1 (*never*) to 6 (*always*).

*Decentering* will be assessed using a brief 5-item version of the decentering subscale of the Toronto Mindfulness Scale [54]. The chosen items are among the highest-loading items on the original subscale. Participants will respond to the following statement—“Please indicate the extent to which each statement describes what you just experienced during the meditation:”—on a 6-point Likert scale ranging from 1 (not at all) to 6 (very much).

*Mindful attitudes* will be assessed using a face-valid selection of (1) Toronto Mindfulness Scale items to reflect attitudes of nonjudgment (acceptance or allowance) and curiosity [54], (2) Five Facet Mindfulness Questionnaire nonjudgment subscale items to reflect nonjudgment (nonevaluative aspect), and (3) Nonattachment Scale items to reflect nonattachment. Participants will respond to the following statement—“Please indicate the extent to which you agree each statement describes what you just experienced during the meditation:”—using a 5-point Likert scale ranging from 1 (not at all) to 5 (very much).

### Participant Timeline

**Figure 2:**
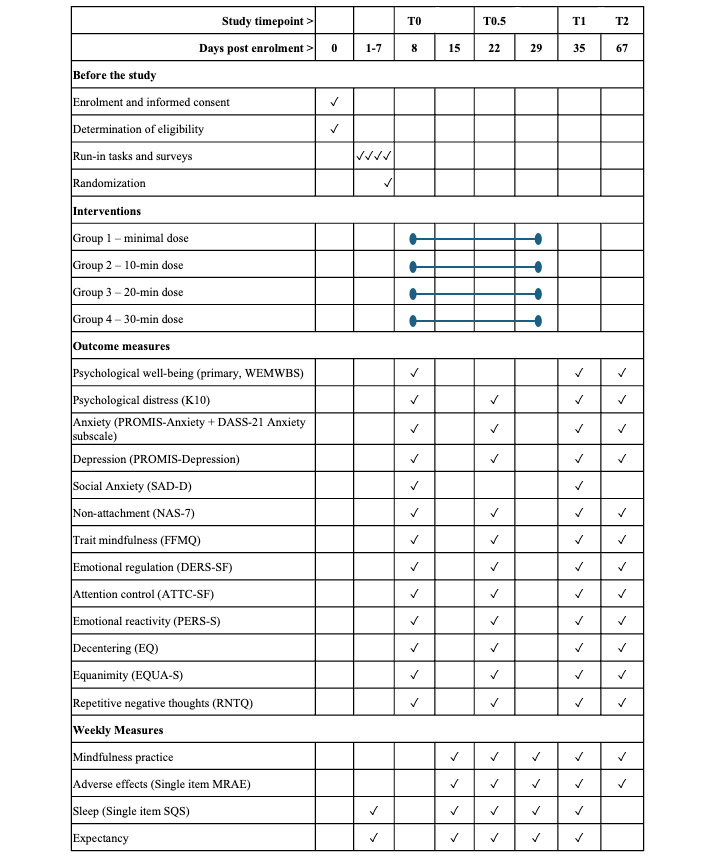
Study timeline. Momentary measures collected daily between T0 and T1 are excluded. T0, T0.5, T1, and T2 represent time points before randomization, midintervention (2 weeks after randomization), postintervention (4 weeks after randomization), and follow-up (8 weeks after randomization). ATTC-SF: Attention Control Scale–Short Form; DASS-21: Depression, Anxiety, and Stress Scale; DERS-SF: Difficulties in Emotion Regulation Scale–Short Form; EQ: Experiences Questionnaire; EQUA-S: Two-Factor Equanimity Scale; FFMQ: Five Facet Mindfulness Questionnaire; K10: Kessler Psychological Distress Scale; MRAE: Meditation-Related Adverse Effects Scale; NAS-7: Nonattachment Scale; PERS-S: Perth Emotional Reactivity Scale–Short Form; PROMIS: Patient-Reported Outcomes Measurement Information System; RNTQ: Repetitive Negative Thoughts Questionnaire; SAD-D: Social Anxiety Disorder Severity Scale; SQS: Sleep Quality Scale; WEMWBS: Warwick-Edinburgh Mental Wellbeing Scale.

A timeline of all major study milestones is provided in Figure 2. For brevity, daily and weekly survey items have been excluded.

### Sample Size

A meta-analysis of meditation apps [1] indicates a standardized mean difference (SMD) effect size of 0.29 (95% CI 0.14-0.45) for psychological well-being when comparing apps to controls. A more recent study [6] randomized participants to approximately 10 or 30 minutes of sitting or movement practice for 2 weeks. They estimated a difference in the effects on the WEMWBS between the sitting groups of short and long duration of SMD=0.2 to 0.25. To be conservative, we determined a target sample size with an SMD of 0.2.

On the basis of an SD in WEMWBS scores of 9.84 [55] and a within-individual correlation of 0.83 in repeated assessments of the WEMWBS [34], we would need a final sample size of 688 participants to achieve 80% power (at the 2-sided *P*=.02 significance level to correct for multiple comparisons between conditions) to detect a difference of 1.97 points (SMD=0.2) for each active condition (10, 20, and 30 min) compared to one another and with the minimal-dose condition. This would translate to approximately 172 participants per arm. These calculations were conducted using the *sampsi* package in Stata (StataCorp).

Meditation app studies indicate overall retention rates of approximately 60% for active mindfulness meditation interventions [1]. Given our use of a run-in period, the aim of which is to allow participants who are most likely to disengage to do so before randomization, we anticipate postrandomization retention rates approximating 80%. In total, we will then recruit on a rolling basis until we achieve the required sample size at randomization of 860.

We will aim to recruit the full sample by March 31, 2025. We will conduct an interim analysis at this stage if the sample is at <90% of the target. If the sample is at ≥90% of the target by March 31, 2025, recruitment will be terminated. If continued recruitment is necessary (ie, we recruit <90% of the sample by March 31, 2025), recruitment will continue until the target sample is reached or 6 months (from March 31, 2025) have elapsed (whichever occurs first). If an interim analysis is required, the team member who undertakes this analysis will not implement the final analysis (as they will need to be unblinded). Other members of the team will undertake the final analysis and will alter the arm designation in the analytic code to further blind results relative to the interim analysis.

### Recruitment

We will recruit participants to the study on a rolling basis via advertisements from the Contemplative Studies Centre using the following streams: trial website, relevant university websites (eg, “volunteer for research” pages), media releases (and media), online advertisements, social media, newsletters, and email lists. We will also promote “snowballing” by sending a flyer through our research, education, practice, media, and advisory networks (and asking them to forward it to their networks) and further asking participants interested or participating in the study to share it on social media and refer a friend. If recruitment is slow, we will additionally consider paid online advertising (eg, through Google or Facebook).

Interested individuals will be directed to the trial website for information about the study, including a plain-language statement about the trial and a link to register interest in participating. The link will take interested individuals to a staged prescreener survey (hosted via Qualtrics) that assesses broad eligibility before looking at specific eligibility to understand the individual’s sociodemographic profile, meditation history, smartphone access and use, and mental health (refer to the Prescreening Measures section). The research team will confirm eligibility before individuals are invited via email to enroll in the study. To accept the invitation, participants will acknowledge that they have read the plain-language summary and will complete an online informed consent form informing them of the aims and particulars of the study and their right to withdraw from it at any time. Once enrolled, participants will receive a welcome email with a link to the first run-in task.

### Assignment of Interventions: Allocation and Blinding

After meeting the selection criteria and completing the run-in period and baseline measures, participants will be randomized to 1 of the 4 groups. Participants will be blinded to the dosing nature of the study and will only be told that they are participating in a trial to test the effects of a 28-day mindfulness meditation intervention. While participants will be informed of their group allocation (eg, group 1), they will not be informed of what the number refers to.

Randomization will be remote and computer based. The allocation process will be automatically concealed, so researchers will not be able to predict or influence who will be randomized to which group. Members of the research team will not be informed of participants’ allocation until after final analyses have been completed, and analyses will be conducted blind to group allocation.

Group allocation will be based on block randomization, with an equal number of participants allocated to each condition for every 24 participants. The randomization process consists of prepopulating a list of randomized allocated group orderings and putting this list in an Amazon Web Services Simple Queue Service First-In-First-Out queue (condition queue), from which group allocation occurs by polling an item from the queue. It is necessary to rebuild this condition queue regularly as queues have a maximum life span of 14 days. We decided to rebuild every night (as opposed to every 14 days), although the rebuilding process maintains the current allocated group orderings from the queue it replaces.

To generate the group ordering (condition list) that is placed on the condition queue, the following steps are taken using a Python script (Python Software Foundation) with the random seed set to 2023: (1) a list with 24 items constituting 1 block is created containing 6 occurrences for each group (ie, 1, 2, 3, 4, 1, 2, 3, 4...1, 2, 3, 4), (2) we randomize this list and then add it to the condition list, (3) this process is repeated 72 times to create a condition list of length 1728 (ie, 72 blocks of 24), and (4) this condition list is uploaded to the condition queue.

This method equates to block randomization as, for every 24 assignments to the condition list, an equal number of participants (ie, 6) will be randomly assigned to each group.

Participants flagged as suspicious and who fail to verify information used to determine their eligibility for the study within the designated time frame (ie, 1 week) will be removed from the study. If removal occurs after randomization, the group to which that participant was allocated will be added to a reassignment queue.

The group assignment for new participants will be based on the next allocation in the reassignment queue. If the reassignment queue is empty, allocation will revert to the condition queue.

### Data Management

Data management and analysis will take place at the Contemplative Studies Centre at the University of Melbourne. During the data collection phase, data from Qualtrics will be encrypted before being stored in their respective cloud-based storage databases. Access to these databases is gated so that entry is only permitted to users entering through the approved route. The research team will access the data via a secure site, which is accessed only using verified credentials, and the data will be stored on a longer-term basis on a password-protected University of Melbourne computer. Qualtrics will be responsible for data storage during the data collection phase. In addition, Unforgettable Research Services Pty Ltd will administer the storage of data on Amazon Web Services. Amazon uses a sophisticated identity management and permission infrastructure that includes rotating keys and encrypted communications. Deidentifiable data will be stored online in perpetuity in line with open science best practices. Storage of downloaded data will be the duty of the responsible researcher, NTVD. Deidentifiable data will be used by the research team for all analyses.

### Statistical Methods

#### General Principles

The primary analysis will be undertaken on the primary outcome following the intention-to-treat approach [56]. The intention-to-treat analysis sample will consist of all randomized participants, assigning them to the arms they were randomized to independently of their subsequent behavior. An intention-to-treat approach was chosen for the primary analysis because the study’s main purpose is to test whether larger *prescribed* doses of mindfulness practice yield larger effects on well-being. Intention-to-treat analyses will also be conducted for all secondary outcomes. Additional secondary analyses will be conducted following the per-protocol approach for all outcome measures (ie, primary and secondary). For data collected at weekly increments between T0 and T1, measurement occasions will be nested within individual participants.

The comparisons of interest will be each intervention (10, 20, and 30 min) compared to one another and with the minimal dose for each outcome (mean difference and 95% CI).

#### Baseline Characteristics

Demographic and baseline variables of participant characteristics will be summarized descriptively and presented by intervention group (4-min active control and 10-, 20-, and 30-min active group). No statistical comparisons between groups will be made at baseline. Characteristics will be summarized using frequencies and percentages (based on the observed sample) for categorical variables, means and SDs for continuous variables, or medians and IQRs for nonnormally distributed continuous variables.

#### Effectiveness Analyses

A constrained longitudinal data analysis method [57] will be used to build the linear mixed-effects model estimating the effect of the interventions on the primary outcome. The model will incorporate study visit (T0 and T1) as a categorical variable, intervention (10, 20, and 30 min each vs the minimal dose), and intervention by study visit interaction as main effects. The model will assume a common baseline mean of the outcome across the 4 intervention groups and an unstructured variance-covariance among the repeated measurements.

Models analyzing secondary outcomes will incorporate study visit (T0, T0.5, and T1) and intervention (10, 20, and 30 min each vs the minimal dose) as categorical variables. Secondary continuous outcomes will be analyzed similarly to the primary outcome (ie, the constrained longitudinal data analysis described previously). Additional analyses will include follow-up assessments (ie, T2) in the model.

#### Efficacy Analyses

Analyses of all outcomes will also be conducted on a per-protocol basis to test the efficacy of different doses under “ideal” conditions of high compliance. The model will incorporate study visit (T0, T0.5, and T1; additional analyses will include T2) and intervention (10, 20, and 30 min each vs the minimal dose) as categorical variables. The per-protocol analysis sample will consist only of those participants who were compliant with the allocated intervention dose, excluding those who did not comply as opposed to reallocating them to an arm representing their actual dose. A “complier” will be defined by having reported session completion for >66.67% and <117% of sessions (100%=6 weekly sessions=24 sessions).

Estimation of effects in randomized controlled trials is known to be biased when there are variable rates of nonadherence to treatment [58]. To control for prerandomization factors that may influence nonadherence to the 10-, 20-, and 30-minute conditions, we will adjust for prerandomization confounders for the per-protocol analysis, such as personality traits (conscientiousness [59,60]), openness to experience [59,61], depressive symptoms [59,61], and motivations and expectations [60]. The method of inverse probability weighting will be used to control for confounding bias due to prerandomization factors [62]. A causal diagram will be drawn to determine a priori potential pre- and postrandomization confounders associated with being a complier [58].

#### Additional Analyses

If any difference between any conditions is observed for any of the primary or secondary analyses, we will explore ways of quantifying that relationship as a means of representing dose-response effects. In particular, we will test for the presence of a linear and log-linear relationship across the treatment doses.

We will describe differential rates of attrition and adherence across the different treatment arms and explore whether any variables collected at baseline predict differential attrition and adherence.

Using estimated practice time per adherence measures, we will implement a receiver operating characteristic analysis to ascertain the practice amount at which individuals achieve a change of ≥7.45 points on the primary outcome of the WEMWBS (estimated temporal SE of measurement of 2.69 [35] using a multiplier of 2.77 [63]) to detect a statistically important change at the individual level.

Further exploratory analyses will be conducted using both the intention-to-treat and per-protocol approaches on outcomes collected at weekly and biweekly intervals.

#### Multiple Testing Adjustment

For the analysis of the primary outcome, we will use a Bonferroni adjustment to control the type I error rate for the primary outcome across all 3 pairwise comparisons by setting the 2-sided significance level at .02. For analyses of secondary outcomes, with nominal α set at *P*<.05, we will adjust for multiple comparisons at the variable level using the false discovery rate procedure [64], which is less conservative than the Bonferroni correction and, therefore, may provide a better balance than Bonferroni between the likelihood of type 1 and type 2 errors.

#### Missing Data Handling

As the primary strategy to handle missing data, the longitudinal constrained analysis assumes that the probability of missing outcome data is not related to the missing data but to some of the observed measured data in the model (missing at random). Our models will use a restricted maximum likelihood estimator to handle missing data. The use of the restricted maximum likelihood estimator will enable us to effectively handle the potential bias introduced by differential attrition as it allows for the estimation of unbiased fixed-effects coefficients and appropriately accounts for the variability introduced by random effects.

#### Sensitivity Analysis

The frequency and percentage of participants with missing data at T0, T0.5, T1, and T2 will be summarized for the primary and secondary outcomes. In addition, baseline and demographic characteristics will be summarized for those with and without missing data for the primary outcome measure (at T0, T0.5, T1, and T2) to explore the missing data assumption and identify and include in the relevant models any variables not included in the target primary analysis that are potentially associated with missingness.

### Monitoring

#### Data Monitoring

In partnership with the trial sponsor, we have determined that the planned work is of low risk and, thus, a data monitoring committee is unneeded. Participant data will be monitored during the study for adverse events and managed by the responsible researcher. If adverse events are severe, an independent data monitoring committee will be formed in consultation with the trial sponsor to evaluate whether the trial needs to be discontinued.

#### Harms

To manage and minimize risks, potential participants will be informed (before consent) of the marginal risk of distress and emotional discomfort when responding to survey questions about symptoms of depression, anxiety, and suicidal ideation. Participants will be informed of their right to withdraw from the study at any point. To address potential distress, in the plain-language statement, we indicate that, should participants experience discomfort or distress because of their participation in the study, they can contact the study lead or one of the provided referral sources. In addition, we will provide details of a range of psychological service providers if they wish not to take up the aforementioned option, including one specifically related to meditation-related difficulties. In the prescreening questionnaire, for any participants who report experiencing mild to severe symptoms of depression or slight to severe symptoms of suicidal ideation, an automated message will appear at the end that refers the participant to psychological support services.

For meditation-related risks, participants will be asked each week whether they have experienced an adverse event during the previous week that they believe may have been caused by their meditation practice. If an adverse event is reported, further screening measures will be administered to assess the nature and severity of the event and whether more follow-up is needed. In such cases in which follow-up is needed, an automated email will be sent to the principal researcher (NTVD), who is a registered psychologist and, therefore, can personally assess what action is required. Adverse event and effect rates will be reported along with the study findings.

#### Auditing

Throughout the data collection phase, the research team will hold regular meetings to manage the project day to day and ensure that all procedures are being followed. The meetings will review recruitment progress, discuss any reported issues by participants, and anticipate and resolve any problems.

### Ethical Considerations

#### Research Ethics Approval

Approval has been obtained from the Human Ethics Team at the University of Melbourne’s Office of Research Ethics and Integrity (23299). The trial protocol has been developed consistent with the SPIRIT (Standard Protocol Items: Recommendations for Interventional Trials) 2013 statement [65]. Results will be reported using the CONSORT-EHEALTH (Consolidated Standards of Reporting Trials of Electronic and Mobile Health Applications and Online Telehealth) clinical trial reporting tool [66].

#### Protocol Amendments

If needed, the study researchers will prepare protocol amendments and will seek approval from the Human Ethics Team at the University of Melbourne. Any updates to the protocol will be reflected in the trial registration on ClinicalTrials.gov, and an updated version will be posted on the Open Science Framework website [22].

#### Consent

The consent form will be embedded within the study landing page in Qualtrics. Participants can provide informed consent to participate in the trial by checking the box at the bottom of the form. Only once informed consent is provided will the participant be able to advance to the prescreening measures.

#### Confidentiality

Data that identify participants will be retained in a separate, password-protected database on an encrypted University of Melbourne computer to which only the responsible researcher will have access. All participants’ data will be stored on a secure server by Unforgettable Research Services Pty Ltd (via Amazon Web Services). Amazon Web Services has extensive security measures, including rotating keys and a comprehensive identity management and permission system. All transmission of information across networks will occur in encrypted form. Only deidentifiable data will be stored online in perpetuity in line with open science best practices. All analyses will be conducted on deidentifiable data.

#### Participant Compensation

To promote study retention, participants will be reimbursed for the completion of surveys at each successive stage of the intervention: Aus $15 (US $9.75) for baseline measures (approximate completion time: 20 min), Aus $5 (US $3.25) for the surveys at the end of week 1 and week 3 (completion time: approximately 3 min each), Aus $15 (US $9.75) for the midintervention survey at the end of week 2 (completion time: approximately 15 min), Aus $1 (US $0.65) for each of the 24 daily surveys (completion time: approximately 24 min), Aus $25 (US $16.24) for postintervention measures (completion time: approximately 20 min), and Aus $20 (US $12.99) for follow-up measures (completion time: approximately 20 min). Total remuneration for participants who complete all surveys is Aus $109 (US $70.82), and they should take approximately 90 minutes to complete over the course of the study. In addition, participants who complete the prescreener will be entered into a draw for 1 of 10 Aus $100 (US $64.97) gift cards via GiftPay. Once eligible and enrolled, participants who successfully complete the run-in period will be entered into a further draw for 1 of 10 Aus $100 (US $64.97) gift cards via GiftPay. Participants will be invited to complete surveys regardless of whether they practice meditation or continue with the course, and no financial reward will be offered for completing meditation practice or listening to the guided practice audio instructions.

### Access to Data

Only researchers listed as authors of this manuscript will have access to the final trial dataset.

### Ancillary and Posttrial Care

The responsible researcher is a registered psychologist who will follow best clinical practices for follow-up and risk management. Given the low-risk nature of the intervention and the prescreening measures, it is not anticipated that additional posttrial care will be required.

### Dissemination Policy

Results will be published in peer-reviewed journals and presented at conferences. A lay summary will be disseminated to a wider audience via the University of Melbourne’s website and promoted via social media.

## Results

This is protocol version 1.1. Version 1.0 was uploaded to the Open Science Framework website in early May 2024 to coincide with the start of recruitment. This study has been prospectively registered at ClinicalTrials.gov with the identifier NCT06378450. Recruitment was paused approximately 6 weeks later after a significant number of participants were identified as being fraudulent and not meeting the eligibility criteria. At this time, <5% of the sample had been recruited. Additional screening procedures have been introduced, and recruitment reopened in October 2024. Version 1.1 of the protocol incorporates these minor changes. We estimate that the results will be published by March 2026.

## Discussion

### Overview

This pragmatic randomized controlled trial aims to conduct a thorough evaluation of the dose-response effects of mindfulness meditation. This will contribute to understanding how much mindfulness practice people need to engage in to promote improved well-being and other psychological outcomes.

The evidence base for dose-response effects in mindfulness meditation practices remains inconclusive. Empirical studies exploring variations in session lengths and frequencies have not consistently demonstrated significant differences in outcomes across different doses [5,7,8]. These studies have had limitations such as small sample sizes and brief overall program durations. Meta-analyses and meta-regression models have also produced mixed findings, with some indicating minor benefits from larger doses over smaller doses and others showing no significant effects [10]. However, these studies lack random allocation of participants to different doses, and therefore, the results may be subject to bias. Overall, current industry practice (advocating for doses as low as 5 min/d) and current research underscore a need for more rigorous studies to establish clearer guidelines on effective mindfulness practice dosages.

### Strengths and Limitations

When compared to previous empirical dose-response assessments, our study has several notable strengths and some limitations. These are described in the following paragraphs.

Our study has several characteristics that will improve our capacity to make inferences about dose-response effects for mindfulness practices compared to previous studies that have tested dose-response effects for mindfulness meditation. Our targeted sample size (N≥866) is powered to detect small effects (*d*=0.2 approximately) between active dose conditions. The program length is 28 days, which will allow us to test for changes in more stable “trait” effects. There is considerable variation in doses for daily sessions, ranging from 10 to 30 minutes. These doses are based on a nonsystematic search of the literature, MBPs, and mindfulness apps with dosage recommendations (for details, refer to the study by Bowles et al [11]). The random allocation of participants to different doses will allow us to make inferences about the effects of different doses, which are less subject to bias than those in previous assessments [3,10]. Finally, while our primary outcome is well-being, we have a range of outcome measures covering different aspects of well-being and mental health, as well as hypothesized mechanisms of change for mindfulness interventions, including decentering and emotional reactivity.

This study is being conducted entirely online. This has the advantage of allowing participants to practice in their homes and other familiar ecologically valid settings, although it also has the limitation of researchers having little control over the research environment. However, a common problem with internet-based interventions is high dropout rates and low adherence. Our primary analysis is to test the effectiveness of different doses, and therefore, our data will be analyzed on an intention-to-treat basis. As such, we are most concerned with the problem of high dropout rates. To minimize dropouts, we are offering financial inducements for participants to continue honestly reporting their practice amount and responding to questionnaires regardless of whether they are still completing the intervention. However, it is still in our interest to have high rates of adherence given that we will also analyze data on a per-protocol basis (ie, as secondary analyses only). To this end, we are using a run-in period to identify and exclude participants who are most likely to drop out and not complete the intervention before randomization. Given this use of the run-in period, we cannot make inferences about the likely adherence to similar mindfulness interventions in real-world settings. Therefore, while the use of a run-in period strengthens internal consistency, it introduces a selection bias. We will attempt to quantify this by reporting sociodemographic data and measures of mental health and well-being for people that complete the run-in period and compare them against those of people who do not. Despite the use of a run-in period, we still anticipate that adherence rates are likely to vary across dosage groups, with lower adherence at higher doses. This may limit our capacity to make inferences about the efficacy of different doses for the per-protocol analyses.

Given our focus on the question of dose-response effects, our analysis examines the effectiveness of different doses relative to each other (and to a minimally active control). Therefore, we cannot make inferences about the effectiveness of the intervention relative to other psychosocial interventions. However, the intervention shares several characteristics that align with the definition of MBPs by Crane et al [30]. Therefore, we are relying on this evidence base, which has demonstrated the effectiveness of similar MBPs, while focusing on one aspect of such programs that is less empirically validated, namely, whether and to what degree shorter or longer daily practices are more beneficial.

All outcome data are self-reported, introducing the potential for recall bias, social desirability bias, and inaccuracies. Despite these limitations, self-report measures were chosen for their practicality and suitability for collecting data online for well-being, the primary outcome, and various secondary outcomes.

Finally, we are tracking and will follow up on reports of adverse experiences throughout the intervention and follow-up periods. Britton [67] has hypothesized that meditation-related adverse experiences and problems with sleep may be more likely to occur at higher doses. While our dosage may still be relatively low compared to that of high-dose settings such as residential retreats, our study design will allow us to explore the question of whether and when higher doses of mindfulness may be riskier than lower doses.

Considering these strengths and limitations, our study will make a significant contribution to the growing literature on the dose-response effects of mindfulness meditation, providing much-needed empirical support to the question of whether and when larger doses are more beneficial than smaller ones. These findings may inform practical guidelines, helping beginner meditators and their advisors—whether in health, well-being, or spiritual contexts—estimate the amount of practice required to achieve particular outcomes.

## Abbreviations

CONSORT-EHEALTH: Consolidated Standards of Reporting Trials of Electronic and Mobile Health Applications and Online Telehealth
MBP: mindfulness-based program
PROMIS: Patient-Reported Outcomes Measurement Information System
SMD: standardized mean difference
SPIRIT: Standard Protocol Items: Recommendations for Interventional Trials
WEMWBS: Warwick-Edinburgh Mental Wellbeing Scale

## Data Availability

Data and other study materials will be openly available on the Open Science Framework website [22].
